# Scientists←Editors←Scientists: The Past, Present, and Future of *PLoS Genetics*

**DOI:** 10.1371/journal.pgen.1000580

**Published:** 2009-07-31

**Authors:** Gregory S. Barsh, Gregory P. Copenhaver

**Affiliations:** 1 Departments of Genetics and Pediatrics, Stanford University School of Medicine, Stanford, California, United States of America; 2 Department of Biology and the Carolina Center for Genome Sciences, The University of North Carolina at Chapel Hill, Chapel Hill, North Carolina, United States of America

*PLoS Genetics* is different: different not only because of the PLoS-wide vision for open access and new ways of communicating science, but also in terms of administration and leadership. We are, first and foremost, a community journal, where editorial decisions and direction are made by consensus. This model, where responsibility is distributed among a team of more than 80 working scientists in a way that promotes and encourages discussion, has been nourished and developed fully by Wayne Frankel, who has been with the journal since its inception, and first introduced us to *PLoS Genetics* exactly four years ago. As the founding Editor-in-Chief, Wayne brought us to where we are today—with nearly 150 new submissions per month, a scope that covers the entire tree of life (and occasionally synthetic biology), and a focus on scientific substance together with a goal of serving the interests of both readers and authors. In making the transition from scientist to Editor-in-Chief, and again to scientist earlier this year, Wayne's contributions have shown how one role can strengthen the other. Happily, he remains an active member of the Editorial Board, shepherding and consulting on manuscripts in the areas of mammalian genetics and neurobiology.

Wayne's scientific career is focused on using genetic approaches in laboratory mice to better understand the causes and pathophysiology of epilepsy. To the extent that phenotype-driven geneticists are fishermen, Wayne has recovered a rich and diverse catch, including several ion channels, nuclear ATPases, and, most recently, RNA binding proteins that regulate a complex set of downstream targets that influence neuronal excitability. From quantitative trait locus analysis to mutagenesis to gene targeting, he has focused on biology rather than technology, maintaining a strong sense of scientific rigor, a healthy scepticism, and a nose for opportunity. Through all of it, Wayne has remained both a community leader and a cutting-edge experimentalist, and we look forward to his continued contributions to mammalian genetics.

Wayne's editorial leadership has helped to move *PLoS Genetics* forward in three important areas. First, by establishing specific sections—Evolution, Natural Variation, and Epigenetics—each with a talented set of senior editors and a common vision, *PLoS Genetics* has managed to be extremely broad but remarkably consistent in its standards and goals. An underlying theme of this organizational structure is that genetics is neither a set of methods and tools, nor a group of scientists organized according to Linnaeus, but instead is a common way of thinking about and approaching biological questions across evolution in which the relationship between genotype (and occasionally epigenotype) and phenotype is paramount.

The foundation of *PLoS Genetics* has been, and will remain, the quality and substance of our Research Articles, but a second area where Wayne's efforts have been apparent is in the journal's development of other article types. We began with what might be considered traditional Reviews, which many journals emphasize as much for their effects on the impact (factor) of the journal as for their utility. But *PLoS Genetics* is different, and with Wayne's leadership, we have gone beyond the traditional Review to feature Perspectives, Jane Gitschier's Interviews (we particularly enjoyed the one with the Honorable Judge John E. Jones, III [1]), and, most recently, Viewpoints, which provide a forum for the discussion of controversial and/or emerging topics of interest to the genetics community.

Third, with the success of the journal over the last four years, Wayne helped *PLoS Genetics* confront both the opportunities and the challenges of growth. Because the PLoS journals are published online, growth is dictated not by traditional concerns of print media—for example, by the number of trees on the planet—but by the science and the genetics community. The commitment of time and expertise on the part of our hard-working Editorial Board (http://www.plosgenetics.org/static/edboard.action) and the community of reviewers who support our peer-review process enables us to keep review and decision-making times as short as possible in the face of rising submissions.

What about the long term? With regard to scientific content, we will follow the course set by Wayne and endorsed by our rich, dynamic, and expanding Editorial Board—emphasizing work of broad interest that provides significant mechanistic insight into a biological process or processes. This means that for the near term, we expect the acceptance rate to remain about the same, ∼30% of all submissions, with an increase over time in the number of articles published each week. It also means that we will continue to rely on Web-based consultation and electronic dialogue among board members to help decide which submissions should proceed through peer review—an approach that makes best use of the review process (and the efforts of our referees), and lets authors know sooner rather than later how well their work matches the scope and significance for the journal as established by our board members, all working scientists themselves. Indeed, an aim of the journal is to blur the distinction between scientist and editor, and we anticipate that an increasing number of authors will be asked to serve as editors in the future. Based on our own experience, serving in one role enhances and enriches the other.

Besides content, what can (and should) *PLoS Genetics* do for scientists? We will aim to explore two areas by the year's end: the way in which publication influences career advancement, and ways in which we can broaden our representation of the global scientific community. Most of us would agree that important career decisions—hiring, promotion, funding—should be based on past performance and future potential to make meaningful contributions. But as highlighted in several venues (including articles from *PLoS Medicine* [2],[3] and, recently, from Mark Johnston, Editor-in-Chief of *GENETICS* [4]), the Thomson Scientific (formerly ISI) impact factor is no longer the only and best metric by which to assess meaningful scientific contributions. As authors, one way to combat the inordinate impact of the impact factor is to submit our best work to journals where our peers play a significant role in editorial decisions. But this tack will be most effective when those same decisions carry significant weight at the hiring and promotion table. From this perspective, we look forward to the development of new tools at all PLoS journals to evaluate article-level metrics, and new ways in which those tools can influence critical steps in scientific career advancement.

Until very recently most scientists in the Western hemisphere concerned themselves primarily with science produced on their own side of the globe—indeed, often only the Northern half. Electronic communication and accelerated economic development has changed not only how cutting-edge science is disseminated but where it is being done. *PLoS Genetics* is perfectly poised to promote this trend toward a more global view of science. Online open access levels the playing field for researchers with limited access to top-quality bricks-and-mortar libraries. Perhaps more importantly, because *PLoS Genetics* isn't anchored to a particular location by a printing press or a geographically localized scientific society, we have the opportunity to include excellent scientists from across the globe in our ranks as editors and contributors. In doing so, we can help shape the evolving global scientific community in ways that embrace the priorities of researchers from all four points of the compass.

In keeping with the path that Wayne set us on, we are committed to maintaining *PLoS Genetics* as the venue of choice for publishing the best in genetics research. At the same time, we are acutely aware that the ways in which science is being practiced, reported, and consumed are rapidly changing. Together, as a scientific community, we can harness those changes and create an exciting future.
